# One-year versus five-year hospital readmission after ischemic stroke and TIA

**DOI:** 10.1186/s12883-019-1242-5

**Published:** 2019-01-29

**Authors:** Anna Therese Bjerkreim, Halvor Naess, Andrej Netland Khanevski, Lars Thomassen, Ulrike Waje-Andreassen, Nicola Logallo

**Affiliations:** 1 Department of Clinical Medicine, University of Bergen, Jonas Lies veg 87, N-5021 Bergen, Norway; 2 Department of Neurology, Haukeland University Hospital, Bergen, Norway; 3 Centre for Age-related Medicine, Stavanger University Hospital, Stavanger, Norway; 4 Norwegian Health Association, Oslo, Norway; 5 Department of Neurosurgery, Haukeland University Hospital, Bergen, Norway

**Keywords:** Hospital readmission, Ischemic stroke, TIA, Risk factors, Epidemiology, Outcome

## Abstract

**Background:**

The burden of hospital readmission after stroke is substantial, but little knowledge exists on factors associated with long-term readmission after stroke. In a cohort comprising patients with ischemic stroke and transient ischemic attack (TIA), we examined and compared factors associated with readmission within 1 year and first readmission during year 2–5.

**Methods:**

Patients with ischemic stroke or TIA who were discharged alive between July 2007 and October 2012, were followed for 5 years by review of medical charts. The timing and primary cause of the first unplanned readmission were registered. Cox regression was used to identify independent risk factors for readmission within 1 year and first readmission during year 2–5 after discharge.

**Results:**

The cohort included 1453 patients, of whom 568 (39.1%) were readmitted within 1 year. Of the 830 patients that were alive and without readmission 1 year after discharge, 439 (52.9%) were readmitted within 5 years. Patients readmitted within 1 year were older, had more severe strokes, poorer functional outcome, and a higher occurrence of complications during index admission than patients readmitted during year 2–5. Cardiovascular comorbidity and secondary preventive treatment did not differ between the two groups of readmitted patients. Higher age, poorer functional outcome, coronary artery disease and hypertension were independently associated with readmission within both 1 year and during year 2–5. Peripheral artery disease was independently associated with readmission within 1 year, and atrial fibrillation was associated with readmission during year 2–5.

**Conclusions:**

More than half of all patients who survived the first year after stroke without any readmissions were readmitted within 5 years. Patients readmitted within 1 year and between years 2–5 shared many risk factors for readmission, but they differed in age, functional outcome and occurrence of complications during the index admission.

## Background

Stroke survivors carry a high risk of new diseases due to functional disability, neurological deficits and pre-existing cardiovascular risk factors and comorbidity [1]. Readmissions after stroke are frequent, particularly during the first 3 months after stroke when more than half of all patients readmitted within 1 year presents [2–4]. The neurological and functional impairments often improve during the first year after stroke, but the number of readmitted patients steadily increases in the chronic phase, and between 67 and 83% of all stroke patients have been readmitted within 5 years [4–6]. Despite this, less is known about the long-term risk of readmission for stroke patients who survive the early phase after stroke without any readmissions.

Several studies have examined predictors for readmission after stroke. Data on causes of readmissions after stroke can be used to prevent future avoidable readmissions. Although the study findings are inconsistent, factors associated with 1-year readmission include older age [2, 7–10], a history of stroke [2], diabetes mellitus, [11] coronary artery disease [2], in-hospital complications [2, 10], longer length of hospital stay [2, 7, 11], and poor functional outcome [2, 11]. Predictors of readmission in the chronic phase of stroke after 1 year have not been identified. Furthermore, it is possible that patients readmitted within the first year differ from patients that experience the first readmission later, as the early readmission may to a higher extent be directly related to acute post-stroke complications and the post-acute stroke treatment, and patients readmitted within the first year may be more severely impaired than patients readmitted later.

The aims of our study were to identify 1) predictors of readmission within 1 year, 2) predictors of first readmission during year 2–5, and 3) factors differing between patients readmitted within 1 year and patients with the first readmission during year 2–5 after ischemic stroke and transient ischemic attack (TIA). We hypothesized that patients readmitted within the first year are more affected by acute complications during the index admission, have more severe strokes and poorer functional outcome than patients readmitted later, and that traditional cardiovascular risk factors are more prominent in patients readmitted within the first year after stroke.

## Methods

All patients > 18 years of age admitted to the Stroke Unit at the Department of Neurology with ischemic stroke or TIA, Haukeland University Hospital from July 1, 2007 to September 31, 2012, were prospectively registered in the Bergen NORSTROKE Registry. The Stroke Unit serves a well-defined geographical area with approximately 275,000 inhabitants. For the present study, we excluded all patients with residence outside the hospital catchment area due to difficulties of long-term follow-up. We also excluded patients who died during the index admission and patients who were discharged to palliative care.

Ischemic stroke was defined as an episode of neurologic deficit lasting > 24 h or clinical symptoms of TIA where computed tomography or magnetic resonance imaging showed acute infarctions related to the initial symptoms [12]. TIA was diagnosed clinically and was defined as transient focal cerebral dysfunction < 24 h with no objective evidence of acute brain infarction on imaging. Duplex ultrasound of the carotid arteries, echocardiogram, Holter monitoring, echocardiography and serology were obtained during hospital admission. Stroke etiology was classified by an experienced stroke neurologist (HN) according to the Trial of Org 10,172 in Acute Stroke Treatment (TOAST) criteria [13]. Stroke severity was determined by the National Institutes of Health Stroke Scale (NIHSS) score on admission and day 7, or at discharge if earlier. Short-term functional outcome was determined by modified Rankin Scale (mRS) and Barthel Index on day 7 or at discharge if earlier. Clinical characteristics, treatment, comorbidity, medical history, in-hospital complications and discharge destination were registered. Secondary prevention was based on the Norwegian guidelines for stroke treatment [14].

Information on readmission and death within 5 years after discharge was collected by review of the medical records from all nine hospitals in the region of the Western Norway Regional Health Authorities. Readmission was defined as the first unplanned admission for any cause to any hospital department after the index admission. The timing and primary cause of the first readmission were registered, and readmitted patients were divided into two groups: first readmission within 1 year after discharge, and first readmission within year 2–5. Causes of readmission were categorized as recurrent stroke, stroke-related event (including neurological deterioration, hemorrhagic transformation of cerebral infarction, neurological complications after carotid endarterectomy, and suspected stroke or TIA with no specific final diagnosis), seizure, cardiac disease, infection, fracture, gastrointestinal hemorrhage, venous thromboembolism, and other cause. Hemorrhagic transformation was defined as petechial or confluent hemorrhage in the same area as the primary infarction.

The study was approved by the Regional Ethics Committee for Medical and Health Research Ethics in Western Norway (REC West). Written informed consent was obtained from all patients. In cases where patients suffered severe strokes and were not able to give informed consent, this was obtained from their legally authorized representatives as required by the Regional Ethics Committee for Medical and Health Research Ethics in Western Norway.

### Statistics

Baseline characteristics were examined by using the χ^2^ test for categorical variables and Student’s *t*-test or Mann-Whitney’s *U*-test for the continuous variables as appropriate. A Kaplan-Meier failure curve was made to demonstrate the incidence of readmission over time, and the log-rank test was used to compare the incidence of readmission between ischemic stroke and TIA. Cox regression analyses were used to examine factors associated with readmission within 1 year and first readmission during year 2–5 after discharge with backward selection method. Patients who were readmitted or dead within 1 year after discharge were excluded from the analyses on readmission from year 2–5. Patients who died or moved outside the area of the Western Norway Regional Health Authorities before the end of follow-up without any readmission were censored in the Kaplan-Meier analysis and the Cox regression models. All multivariate analyses were performed with stepwise backward elimination starting with all significant parameters from the univariate analyses (*P* < .05). Age, sex and mRS score were forced into each analysis to adjust for potential confounding. Statistical analyses were performed using Stata 14.0 (Stata Corporation, College Station, TX, USA).

## Results

The study cohort included 1453 patients discharged alive from our stroke unit, of whom 1303 (89.7%) were diagnosed with ischemic stroke and 150 (10.3%) were diagnosed with TIA. Baseline characteristics of the study cohort are shown in Table 1.

**Table 1:** Baseline characteristics of the study cohort

Characteristics	Values
Age (years), mean ± SD	73.1 ± 13.7
Male sex, N (%)	782 (53.8)
NIHSS score at discharge, median (IQR)	1 (0, 4)
mRS score at discharge, median (IQR)	2 (1, 3)
Barthel Index at discharge, median (IQR)	100 (70, 100)
Stroke subtype, N (%)	
Large-artery atherosclerosis	193 (13.3)
Cardioembolism	473 (32.6)
Small vessel occlusion	159 (10.9)
Other determined etiology	20 (1.4)
Undetermined etiology	608 (41.8)
Comorbidity, N (%)	
Prior stroke	299 (20.6)
Peripheral artery disease	102 (7.0)
Coronary artery disease	325 (22.4)
Diabetes mellitus	211 (14.5)
Atrial fibrillation	428 (29.5)
Hypertension	813 (56.0)
Ever-smoker	832 (57.3)
Complications during index admission, N (%)	
Pneumonia	112 (7.7)
Enteral feeding	117 (8.1)
Urinary tract infection	184 (12.7)
Urinary retention	327 (22.5)
Urinary incontinence	212 (14.6)
Seizures	35 (2.4)
Stroke in progression	130 (9.0)
Any complication	599 (41.2)
Length of stay, median (IQR)	6 (3, 11)
Treatment at discharge, N (%)	
Antiplatelet	917 (63.1)
Anticoagulation	465 (32.0)
Antihypertensive	1021 (70.3)
Statins	1023 (70.4)
Discharge status, N (%)	
Home without care	792 (54.6)
Home with care	164 (11.3)
Rehabilitation department	112 (7.7)
Nursing home	337 (23.2)
Other department	46 (3.2)

**Fig. 1:**
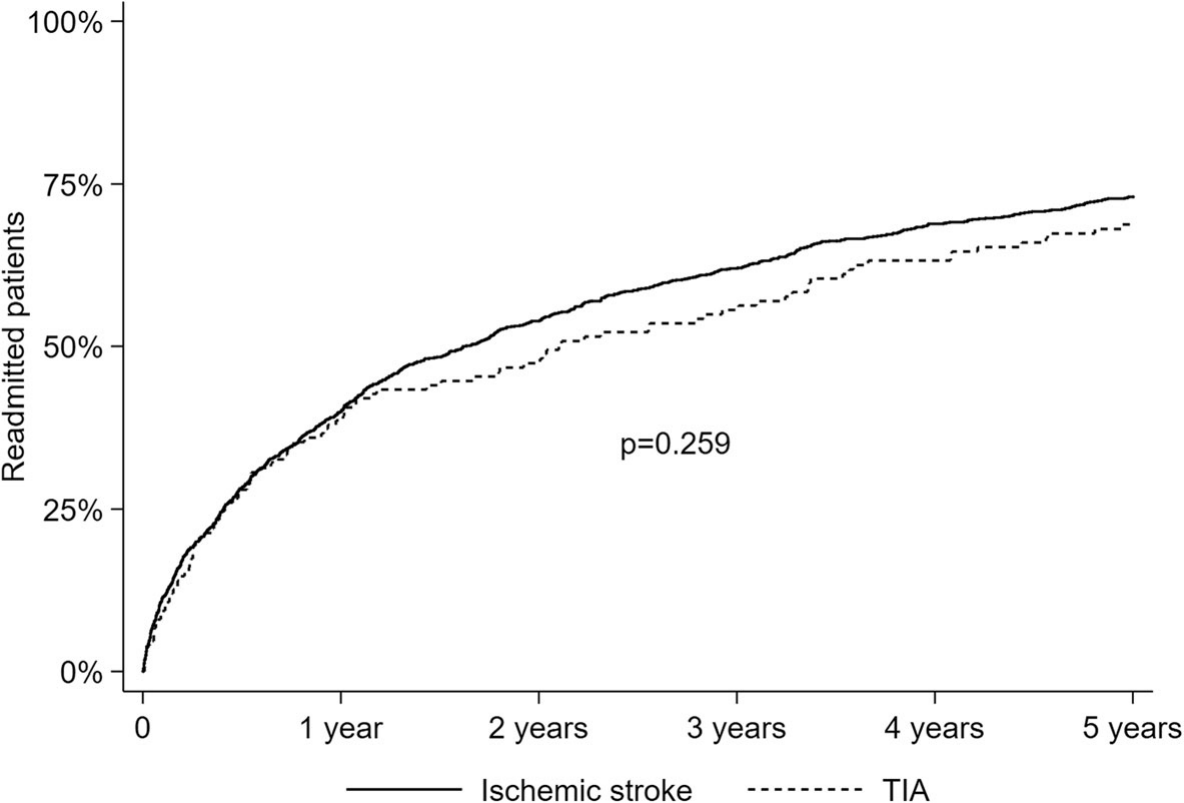
Kaplan Meier failure curves showing incidence estimates of readmission within 5 years for patients with ischemic stroke (*n* = 1303) or TIA (*n* = 150)

Within 1 year after discharge from the index admission, 568 (39.1%) patients were readmitted and 163 (11.2%) patients were dead, of whom 108 (66.3%) had been readmitted at least once. Of the 830 patients that were alive and free of readmission 1 year after discharge, 439 (52.9%) were readmitted within 5 years. Figure 1 demonstrates the incidence estimates of readmission over time. There were no differences in the 5-year incidence of readmission between ischemic stroke and TIA. After 5 years, a total of 1007 patients (69.3%) had been readmitted, and 358 patients (24.6%) were alive and without any readmission. A total of 492 patients (33.9%) were dead wihtin 5 years after discharge, of whom 280 (56.9%) had been readmitted within 1 year and 124 (25.2%) had the first readmission during year 2–5.

Baseline characteristics of patients readmitted within 1 year and patients with the first readmission between year 2–5 are shown in Table 2. On univariate analysis, patients readmitted within 1 year were older, had a poorer short-term functional outcome, a higher discharge NIHSS score, and more complications during index admission than patients with the first readmission between year 2–5. A stroke subtype of small vessel occlusion was more frequent in patients with the first readmission between year 2–5. Cardiovascular risk factors and comorbidity did not differ between the two groups of readmitted patients, and neither did prescribed secondary preventive treatment at discharge or at the time of the first readmission. A higher percentage of patients readmitted within the first year had stopped smoking before the index admission, whereas more patients had quit smoking after the index admission in patients that were readmitted between years 2–5. Of the patients that were smoking at the time of the index stroke, 37% of the patients readmitted between year 2–5 had stopped smoking compared to 28% of patients readmitted during the first year (*p* = 0.144). Patients readmitted within 1 year were more often discharged to nursing homes and less often discharged home without help than patients readmitted between year 2–5.

**Table 2:** Distribution of baseline characteristics between patients readmitted within 1 year and patients with the first readmission between year 2–5

	≤1 year *N* = 568	Year 2–5 *N* = 439	*P*
Age (years), mean ± SD	76.2 ± 12.5	73.3 ± 13.1	< 0.001
Male sex, N (%)	298 (52.5)	242 (55.1)	0.401
NIHSS score at discharge, median (IQR)	2 (0, 5)	1 (0, 3)	< 0.001
mRS score at discharge, N (%)			< 0.001
0–2	323 (56.9)	304 (69.3)	
3–5	245 (43.1)	135 (30.8)	
Barthel Index at discharge, median (IQR)	100 (60, 100)	100 (85, 100)	< 0.001
Stroke diagnosis, N (%)			0.992
Ischemic stroke	510 (89.8)	395 (90.0)	
TIA	58 (10.2)	44 (10.0)	
Stroke subtype, N (%)			0.070
Large-artery atherosclerosis	85 (15.0)	53 (12.1)	
Cardioembolism	206 (36.3)	152 (34.6)	
Small vessel occlusion	44 (7.8)	55 (12.5)	
Other determined etiology	8 (1.4)	3 (0.7)	
Undetermined etiology	225 (39.6)	176 (40.1)	
Comorbidity, N (%)			
Prior stroke	137 (24.1)	96 (21.9)	0.401
Peripheral artery disease	54 (9.5)	29 (6.6)	0.097
Coronary artery disease	162 (28.5)	103 (23.5)	0.071
Diabetes mellitus	97 (17.1)	64 (14.6)	0.283
Atrial fibrillation	197 (34.7)	135 (30.8)	0.188
Hypertension	358 (63.0)	256 (58.3)	0.128
Ever-smoker	384 (67.6)	285 (64.9)	0.371
Complications during index admission, N (%)			
Pneumonia	54 (9.5)	22 (5.0)	0.007
Enteral feeding	59 (10.4)	24 (5.5)	0.005
Urinary tract infection	86 (15.1)	49 (11.2)	0.066
Urinary retention	154 (27.1)	76 (17.3)	< 0.001
Urinary incontinence	107 (18.8)	47 (10.7)	< 0.001
Seizures	17 (3.0)	9 (2.1)	0.350
Stroke in progression	59 (10.4)	25 (5.7)	0.008
Any complication	281 (49.5)	157 (35.8)	< 0.001
Length of stay, median (IQR)	6 (3, 11)	6 (3, 10)	0.190
Discharge status, N (%)			< 0.001
Home without care	257 (45.4)	255 (58.1)	
Home with care	84 (14.8)	57 (13.0)	
Rehabilitation department	37 (6.5)	35 (8.0)	
Nursing home	162 (28.6)	83 (18.9)	
Other department	26 (4.6)	9 (2.1)	
Treatment at discharge, N (%)			
Platelet inhibitor	379 (66.7)	302 (68.8)	0.487
Anticoagulation	203 (35.7)	148 (33.7)	0.805
Antihypertensive drug	363 (63.9)	285 (64.9)	0.740
Statins	390 (68.7)	322 (73.4)	0.105
Treatment at readmission, N (%)			
Platelet inhibitor	368 (64.8)	281 (64.0)	0.798
Anticoagulation	211 (37.2)	151 (34.4)	0.367
Antihypertensive drug	371 (65.3)	286 (65.2)	0.956
Statins	365 (64.3)	267 (60.8)	0.263
Smoking status at readmission, N (%)			0.036
Stopped smoking before index stroke	242 (66.1)	160 (58.0)	
Stopped smoking after index stroke	35 (9.6)	43 (15.6)	
Still smoking at readmission	89 (24.3)	73 (26.5)	

Increasing age, poorer short-term functional outcome (increasing mRS score), and a history of peripheral artery disease, coronary artery disease, or hypertension, independently increased the risk of readmission within 1 year after discharge, whereas a stroke subtype of small vessel occlusion significantly decreased the risk of readmission within 1 year (Table 3). Increasing age, poorer short-term functional outcome, a history of coronary artery disease, atrial fibrillation, or hypertension, independently increased the risk of readmission between year 2–5 if alive and free of readmission 1 year after discharge (Table 4).

**Table 3:** Risk factors for readmission within 1 year after ischemic stroke or TIA

	Hazard Ratio	95% Confidence Interval	*P*
Age, years	1.02	1.01–1.03	<.001
Male sex	1.02	0.86–1.21	.835
mRS score	1.15	1.09–1.22	<.001
Small vessel occlusion	0.67	0.49–0.91	.010
Peripheral artery disease	1.42	1.06–1.89	.018
Coronary artery disease	1.29	1.07–1.57	.009
Hypertension	1.26	1.05–1.50	.012

**Table 4:** Risk factors for readmission between year 2-5 if alive and without readmission 1 year after ischemic stroke or TIA

	Hazard Ratio	95% Confidence Interval	P
Age, years	1.03	1.02–1.04	<.001
Male sex	1.13	0.93–1.38	.217
mRS score	1.09	1.02–1.16	.014
Coronary artery disease	1.50	1.20–1.89	<.001
Atrial fibrillation	1.25	1.01–1.55	.046
Hypertension	1.38	1.13–1.68	.001

The most frequent cause of the first readmission within 1 year was infection, followed by recurrent stroke, stroke-related event and cardiac disease In contrast, cardiac disease was the most frequent cause of the first readmission for patients readmitted during years 2–5, followed by infection, stroke-related event and recurrent stroke (Table 5).

**Table 5:** Primary cause of the first readmission after ischemic stroke or TIA

	≤ 1 year *N* = 568		Year 2–5 *N* = 439	
	*N*	%	*N*	%
Recurrent stroke	72	12.7	36	8.2
Ischemic stroke	59	10.4	25	5.7
Intracerebral hemorrhage	6	1.1	5	1.1
TIA	7	1.2	6	1.4
Stroke-related events^a^	72	12.7	45	10.3
Seizures	19	3.4	4	0.9
Heart disease	62	10.9	75	17.1
Myocardial infarction	16	2.8	23	5.2
Arrhythmias	17	3.0	22	5.0
Heart failure	13	2.3	12	2.7
Other	16	2.8	18	4.1
Infections	107	18.8	70	16.0
Pneumonia	46	8.1	25	5.7
Urinary tract infection	24	4.2	15	3.4
Sepsis	11	1.9	4	0.9
Other	26	4.6	26	5.9
Fractures	37	6.5	33	7.5
Hip fracture	19	3.3	16	3.6
Other	18	3.2	17	3.9
Gastrointestinal hemorrhage	14	2.5	6	1.4
Venous thromboembolism	4	0.7	3	0.7
Other causes	181	31.9	167	38.0

^a^Neurological deterioration, hemorrhagic transformation of cerebral infarction, neurological complications after carotid endarterectomy, and suspected stroke or TIA with no specific final diagnoses

## Discussion

We found that 69.3% were readmitted within 5 years after ischemic stroke or TIA, with more than half of all readmitted patients presenting during the first year. A smaller Norwegian study demonstrated that 56% of all readmissions within 10 years presented during the first year after stroke and similar patterns have been reported from the US [4–6]. In line with previously reported 1-year readmission rates between 31 and 55%, we found that 39% were readmitted within the first year after discharge [2, 3, 6, 15, 16]. The high rates of readmission during the first year after stroke might reflect an increased vulnerability for post-stroke complications such as infections, recurrent stroke and other cardiovascular events in the early phase after stroke [1].

We also found that more than half of all patients that survived for 1 year without any readmission were readmitted within 5 years. The frequency of the different causes of readmission varied slightly depending on the timing of the first readmission, but infection, cardiac disease, recurrent stroke and stroke-related events were the four most common causes in both time periods. Higher age, poor functional outcome, hypertension and coronary artery disease were all independently associated with readmission both within 1 year and during year 2–5. This indicates that even if the patients survive the vulnerable early period after stroke, they still have a high risk of readmission for the same causes as patients readmitted during the first year after stroke, and the same factors might impact the risk of readmission also in the more chronic phase after stroke.

Higher age and poorer short-term functional outcome predicted readmission within 1 year and from year 2-5, but patients readmitted within 1 year were older, had a poorer short-term functional outcome and more complications during index admission than patients readmitted later. Complications during the index stroke admission have been associated with early readmission in several studies, and probably relates to both high age and poor functional outcome [17, 18]. Higher age and poor functional outcome have been identified as predictors for readmission after stroke in several other studies [2, 7–11]. Although age is a non-modifiable risk factor for readmission, a focus on improving and maintaining physical function and mobility during the stroke hospitalization and in the period after discharge for patients at all ages is important and could potentially help prevent some readmissions.

Hypertension and coronary artery disease were identified as common predictors of readmission within 1 year and from year 2-5, and contrary to our hypothesis, the vascular risk factor profile was not more prominent in patients readmitted within 1 year. Hypertension and coronary artery disease are important risk factors for recurrent stroke and cardiac disease, which were frequent causes of readmission within both 1 year and year 2-5 in our study, and are highly important causes of death after stroke [19, 20]. Hypertension is the most important modifiable risk factor for stroke and other cardiovascular diseases, and blood pressure reduction reduces the risk of recurrent stroke, myocardial infarction and cardiovascular death after stroke [21]. This emphasizes the importance of accurate and aggressive treatment of cardiovascular risk factors in preventing new vascular events and vascular mortality after ischemic stroke and TIA.

Even though cardiovascular risk factors did not differ significantly between patients readmitted within 1 year and patients readmitted during year 2–5, recurrent stroke and stroke-related events were more frequent causes of readmission within 1 year, whereas cardiac disease was a more frequent cause of readmission for patients that were readmitted during year 2–5. We have only included the cause of the first uplanned readmission in this study, but studies have shown similar results in relation to recurrent stroke and cardiac disease, with the highest incidences of recurrent stroke observed in the early period after stroke, and lower, yet more stable, yearly incidences of myocardial infarction and congestive heart failure [4, 22].

Even though they differ in functional outcome, ischemic stroke and TIA patients did not differ in the incidence of readmission. Compared to ischemic stroke patients, both lower, similar and higher rates of readmission in TIA patients have been reported [8, 23–25]. This could possibly be explained by differences in case mix and use of secondary prevention and adherence to medical treatment [25]. Nevertheless, TIA patients have the same risk factor profile and underlying causes of their ischemic event as patients with ischemic stroke, and should be provided accurate secondary treatment in order to reduce subsequent vascular events and readmissions.

Our study has some limitations. Some patients may have been readmitted to a hospital outside the region of the Western Norwegian Regional Health authorities, even though this region involves a large geographical area of Western Norway. Furthermore, we have no information on smoking status or medical treatment received after discharge in patients that were not readmitted, and no information on treatment compliance. As the study cohort originates from a single site, the generalizability of our findings may be limited. A strength of our study is the ascertainment of data related to the readmissions made by review of medical records, and the inclusion of a relatively large study population investigated in a single stroke center according to a predefined protocol with comprehensive data collection. Although other studies have reported resembling results regarding rates and causes of long-term readmission after stroke, our study elaborates on existing knowledge by describing rates, causes and predictors of readmission for patients who survive the first year after stroke without any readmission.

## Conclusions

More than half of all patients who survived the first year after stroke without any readmission, were readmitted within 5 years. Patients with the first readmission during year 2–5 after discharge shared several risk factors with patients readmitted within 1 year, but they differed in relation to short-term functional outcome, age, and the frequency of complications during index admission. Our results indicate that further improvement in the effort of reducing post-stroke complications and hospital readmissions should continue beyond the acute phase after stroke. More studies are needed to establish predictors of readmission after stroke in the long-term perspective.

## Abbreviations

IQR: Interquartile range
mRS: Modified Rankin scale
NIHSS: National Institute of Stroke Scale
SD: Standard deviation
TIA: Transient ischemic attack
TOAST: Trial of Org 10,172 in Acute Stroke Treatment
